# Association Between Serum Carcinoembryonic Antigen Levels at Different Perioperative Time Points and Colorectal Cancer Outcomes

**DOI:** 10.3389/fonc.2021.722883

**Published:** 2021-10-08

**Authors:** Zhenhui Li, Dafu Zhang, Xiaolin Pang, Shan Yan, Ming Lei, Xianshuo Cheng, Qian Song, Le Cai, Zhuozhong Wang, Dingyun You

**Affiliations:** ^1^ Department of Radiology, The Third Affiliated Hospital of Kunming Medical University, Yunnan Cancer Hospital, Yunnan Cancer Center, Kunming, China; ^2^ Department of Radiotherapy, The Sixth Affiliated Hospital of Sun Yat-sen University, Guangzhou, China; ^3^ Yunnan Key Laboratory of Stem Cell and Regenerative Medicine, Biomedical Engineering Research Center, Kunming Medical University, Kunming, China; ^4^ Department of Clinical Laboratory Medicine, The Third Affiliated Hospital of Kunming Medical University, Yunnan Cancer Hospital, Yunnan Cancer Center, Kunming, China; ^5^ Department of Colorectal Surgery, The Third Affiliated Hospital of Kunming Medical University, Yunnan Cancer Hospital, Yunnan Cancer Center, Kunming, China; ^6^ Cancer Research Institute, The Third Affiliated Hospital of Kunming Medical University, Yunnan Cancer Hospital, Yunnan Cancer Center, Kunming, China; ^7^ School of Public Health, Kunming Medical University, Kunming, China; ^8^ The Key Laboratory of Myocardial Ischemia, Harbin Medical University, Ministry of Education, Harbin, China; ^9^ Cardiology Division, The Second Affiliated Hospital of Harbin Medical University, Harbin, China

**Keywords:** colorectal cancer, carcinoembryonic antigen, adjuvant chemotherapy, recurrence risk, risk stratification

## Abstract

**Background:**

Whether elevated postoperative serum carcinoembryonic antigen (CEA) levels are prognostic in patients with stage II colorectal cancer (CRC) remains controversial.

**Patients and Methods:**

Primary and sensitivity analysis populations were obtained from a retrospective, multicenter longitudinal cohort including consecutive patients without neoadjuvant treatment undergoing curative resection for stage I–III CRC. Serum CEA levels before (CEA_pre-m1_) and within 1 (CEA_post-m1_), 2–3 (CEA_post-m2–3_), and 4–6 months (CEA_post-m4–6_) after surgery were obtained, and their associations with recurrence-free survival (RFS) and overall survival (OS) were assessed using Cox regression. Sensitivity and subgroup analyses were performed.

**Results:**

Primary and sensitivity analysis populations included 710 [415 men; age, 54.8 (11.6) years] and 1556 patients [941 men; age, 56.2 (11.8) years], respectively. Recurrence hazard ratios (HRs) in the elevated CEA_pre-m1_, CEA_post-m1_, CEA_post-m2–3_, and CEA_post-m4–6_ groups were 1.30 (95% CI: 0.91–1.85), 1.53 (95% CI: 0.89–2.62), 1.88 (95% CI: 1.08–3.28), and 1.15 (95% CI: 0.91–1.85), respectively. The HRs of the elevated CEA_pre-m1_, CEA_post-m1_, CEA_post-m2–3_, and CEA_post-m4–6_ groups for OS were 1.09 (95% CI: 0.60–1.97), 2.78 (95% CI: 1.34–5.79), 2.81 (95% CI: 1.25–6.30), and 3.30 (95% CI: 1.67–.536), respectively. Adjusted multivariate analyses showed that both in the primary and sensitivity analysis populations, elevated CEA_post-m2–3_, rather than CEA_pre-m1_, CEA_post-m1_, and CEA_post-m4–6_, was an independent risk factor for recurrence, but not for OS. The RFS in the elevated and normal CEA_post-m2–3_ groups differed significantly among patients with stage II disease [n = 266; HR, 2.89; 95% CI, 1.02–8.24 (primary analysis); n = 612; HR, 2.69; 95% CI, 1.34–5.38 (sensitivity analysis)].

**Conclusions:**

Elevated postoperative CEA levels are prognostic in patients with stage II CRC, with 2–3 months after surgery being the optimal timing for CEA measurement.

## Introduction

Colorectal cancer (CRC) is the third leading cause of cancer-related death both in men and women worldwide (1). Tumor relapse is the primary cause of poor prognosis in patients with CRC (2). Predicting the risk of relapse could allow a more targeted approach with respect to the selection of adjuvant therapies and follow-up strategies (e.g., by defining subgroups) for improving overall survival (3).

Carcinoembryonic antigen (CEA) is regarded as an essential indicator of CRC prognosis (4), and the guidelines recommend that serum CEA should be measured preoperatively and postoperatively in patients with CRC (5–9). Recent studies confirm that the preoperative and postoperative serum CEA levels are both associated with CRC outcomes, and elevated postoperative CEA levels are more prognostic than elevated preoperative CEA levels (4, 10–16). Hence, routine measurement of postoperative CEA levels is warranted.

Whether elevated postoperative CEA levels are prognostic in patients with stage II CRC remains controversial (4, 10–13). Some studies report that postoperative CEA levels have a predictive value in patients with stage II CRC (11, 12), while several others have been unable to determine the significance of postoperative CEA levels in such patients (4, 10, 13). A systematic review of published studies (4, 10–13) showed that the time points of postoperative CEA measurement varied across studies. CEA was measured within 4–12 weeks after surgery in some studies (11, 12) and within 1–12 weeks after surgery in several others (4, 10, 13). The difference in the time points of postoperative CEA measurement may be responsible for the inconsistent results, and the optimal timing for postoperative serum CEA measurement is therefore unknown.

In this study, we aimed to examine the association between serum CEA levels at different perioperative time points and CRC outcomes using a retrospective, multicenter longitudinal cohort and to determine the optimal timing for postoperative serum CEA measurement.

## Patients and Methods

### Ethics Approval and Informed Consent

The ethics committee of each participating hospital approved this multicenter retrospective study. The requirement for informed consent was waived by the board, owing to the study’s retrospective nature. All the patient data in the survey were anonymized. This study followed the Strengthening the Reporting of Observational Studies in Epidemiology (STROBE) reporting guidelines.

### Patients

**Figure 1:**
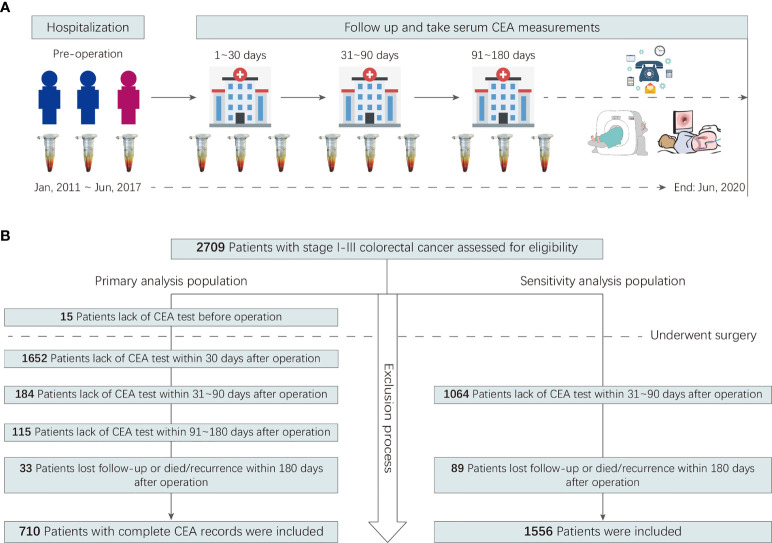
Study flowchart.

A multicenter retrospective cohort was created. It included all consecutive patients with CRC who did not receive neoadjuvant treatment but underwent curative resection for stage I–III colorectal adenocarcinoma between January 2011 and June 2017 at two hospitals in China. A detailed description of the cohort’s inclusion and exclusion criteria can be found in the **Online-Only Supplement**. Participants were included in the primary analysis population if preoperative serum CEA data and postoperative serum CEA measurements obtained within 1, 2–3, and 4–6 months after surgery were available. Participants were included in the sensitivity analysis population if postoperative serum CEA measurements obtained within 2–3 months after surgery were available. The study flowchart is shown in **Figure 1**.

### Serum CEA Determination

Preoperative serum CEA level (CEA_pre-m1_) was defined as the CEA level obtained closest to the time of surgery (as long as it was obtained within 4 weeks before surgery). Postoperative serum CEA level was defined as the last CEA value obtained 1 (CEA_post-m1_), 2–3 (CEA_post-m2–3_), and 4–6 months (CEA_post-m4–6_) after surgery (a month was defined as 30 natural days). The CEA status was classified into two types as follows: normal (≤5.0 ng/mL) and elevated (>5.0 ng/mL). All CEA measurements were made with a chemiluminescence immunoassay using the Cobas 8000 e602 immunoassay analyzer (Roche Diagnostics, Tokyo, Japan) at Yunnan Cancer Hospital and an Alinity i immunoassay analyzer (Abbott Diagnostics, Chicago, IL, USA) at The Sixth Affiliated Hospital of Sun Yat-sen University, following World Health Organization standard methods (code 73/601) (17).

### Surveillance Protocol and Outcome

The surveillance protocol was detailed in our previous study (18). In this study, follow-up ended on June 30, 2020. The primary outcome was recurrence-free survival (RFS). Recurrence included local recurrence and distant metastases, which were confirmed *via* a biopsy sample, positive imaging findings, or histological analyses. RFS was calculated from the date of surgery until the date of recurrence, death, or last follow-up. Data from patients who died or were lost to follow-up were treated as censored. The secondary outcome was overall survival (OS).

### Covariates

Covariates included age, sex, surgical approach (open resection or laparoscopic resection), primary site, tumor differentiation, tumor–node–metastasis (TNM) stage (I-III), lymph node yield (yes or no), mucinous (colloid) type (yes or no), the presence of lymphovascular invasion (yes or no), the presence of perineural invasion (yes or no), and the use of adjuvant chemotherapy (yes or no).

### Statistical Analysis

All statistical analyses were performed using R (version 3.6.2). All tests were 2-sided, and *P* values <.05 indicated statistical significance. The mean, standard deviation (SD), and minimum and maximum values were used to describe results for continuous variables with a normal distribution (including age and body mass index [BMI]); these were further compared using the independent two-sample *t*-test. The group-specific number and percentage of patients in each category were used to describe results for categorical parameters, which were further compared using the chi-square (χ^2^) test.

Differences in RFS between normal and elevated CEA groups at different time points were assessed using the Cox proportional hazards regression model. Hazard ratios (HRs) with two-sided 95% confidence intervals (CIs) were calculated for each group. Cumulative event curves were used to demonstrate the 3-year recurrence of patients with CRC, and log-rank tests were utilized to statistically analyze the differences between the two CEA groups.

To test the robustness of the risk estimates, we used two additional sensitivity analyses. (1) Multivariate Cox proportional hazards regression analysis with stepwise variable selection was performed to identify independent risk factors for recurrence and death. Three models were used: model 1 was unadjusted and constructed using CEA_pre-m1_, CEA_post-m1_, CEA_post-m2–3,_ and CEA_post-m4–6_; model 2 was a version of model 1 adjusted for demographic variables; and model 3 was a version of model 2 adjusted for clinicopathological variables as well. (2) The statistical analyses used in the primary population were also performed in the expanded sensitivity analysis population.

To test for potential sources of heterogeneity, subgroup analyses were performed after stratification by age, sex, BMI, primary tumor site, tumor differentiation, mucinous (colloid) type, cancer stage, lymph node yield, the presence of lymphovascular invasion, the presence of perineural invasion, tumor deposit, CEA_pre-m1,_ and CEA_post-m1_, with tests for interaction using the Cox regression model. Forest charts of subgroup-stratified analyses were created using the R package “forestplot.”

To distinguish between high-recurrence risk and low-recurrence risk patients, associated with RFS differences, we have used maximally selected rank statistics to determine the potential threshold value of CEA (19).

## Results

### Patient Characteristics

In total, 710 patients were included in the primary analysis. The number of participants assessed for eligibility and the reasons for exclusion are shown in **Figure 1**. The 710 patients included 415 men (58.5%), and the mean (SD) age was 54.8 (11.6) years. The mean age and SD of female and male patients were 54.2 ± 11.2 and 55.3 ± 11.8 years, respectively. The 385 patients underwent laparoscopic surgery, 325 underwent open surgery. A total of 699 (98.5%) patients had adjuvant chemotherapy. The median long-term follow-up duration was 49.0 [interquartile range (IQR): 38.7–66.6] months. During the follow-up period, 152 patients (21.4%) showed recurrence, with an incidence density of 24.7 per 1,000 person-years. The characteristics of the primary analysis population are shown in **Table 1**.

**Table 1:** Demographic and Clinicopathological Characteristics of Primary Analysis Population.

Characteristics	Total (*N* =710)	CEA _post-m2-3_		*P v*alue
		≤5 ng/ml (*n* = 662)	>5 ng/ml (*n* = 48)	
Age, year				
Mean (SD)	54.9 (11.6)	54.7 (11.6)	57.8 (11.0)	0.06
Range	(18.0-86.0)	(18.0-86.0)	(34.0-76.0)	
Sex, no. (%) of patients				
Male	415 (58.5)	385 (58.2)	30 (62.5)	0.66
Female	295 (41.5)	277 (41.8)	18 (37.5)	
BMI^a^				
Mean (SD)	23.0 (3.1)	23.0 (3.1)	22.9 (3.5)	0.87
Range	(15.2-35.4)	(16.8-35.4)	(15.2-29.8)	
Primary site, no. (%) of patients				
Colon	463 (65.2)	428 (64.7)	35 (72.9)	0.32
Rectum	247 (34.8)	234 (35.3)	13 (27.1)	
Pathological stage, no. (%) of patients				
I	22 (3.1)	21 (3.2)	1 (2.1)	0.25
II	266 (37.5)	253 (38.2)	13 (27.1)	
III	422 (59.4)	388 (58.6)	34 (70.8)	
Tumor differentiation, no. (%) of patients				
Well	29 (4.1)	28 (4.2)	1 (2.1)	0.25
M oderate	463 (65.2)	426 (64.4)	37 (77.1)	
Poor	197 (27.7)	189 (28.5)	8 (16.7)	
Unknown	21 (3.0)	19 (2.87)	2 (4.2)	
Mucinous (colloid) type, no. (%) of patients^a^				
Yes	39 (5.5)	36 (5.4)	3 (6.3)	>0.99
No	671 (94.5)	626 (94.6)	45 (93.8)	
T stage, no. (%) of patients				
T1 & T2	59 (8.3)	55 (8.3)	4 (8.3)	0.26
T3	592 (83.4)	555 (83.8)	37 (77.1)	
T4	59 (8.3)	52 (7.9)	7 (14.6)	
N stage, no. (%) of patients				
N0	287 (40.4)	274 (41.4)	13 (27.1)	0.15
N1	289 (40.7)	265 (40.0)	24 (50.0)	
N2	134 (18.9)	123 (18.6)	11 (22.9)	
Lymph node yield, no. (%) of patients^a^				
<12	105 (14.8)	101 (15.3)	4 (8.3)	0.27
≥12	605 (85.2)	561 (84.7)	44 (91.7)	
Lymphovascular invasion, no. (%) of patients				
Yes	96 (13.5)	86 (13.0)	10 (20.8)	0.19
No	614 (86.5)	576 (87.0)	38 (79.2)	
Perineural invasion, no. (%) of patients^a^				
Yes	68 (9.6)	60 (9.1)	8 (17.0)	0.13
No	641 (90.4)	602 (90.9)	39 (83.0)	
Tumor deposit, no. (%) of patients^a^				
Positive	55 (11.7)	50 (11.3)	5 (18.5)	0.41
Negative	416 (88.3)	394 (88.7)	22 (81.5)	
Adjuvant chemotherapy, no. (%) of patients				
Yes	699 (98.5)	652 (98.5)	47 (97.9)	0.54^b^
No	11 (1.5)	10 (1.5)	1 (2.1)	
Adjuvant radiotherapy, no. (%) of patients				
Yes	6 (0.8)	5 (0.8)	1 (2.1)	0.34^b^
No	704 (99.2)	657 (99.2)	47 (97.9)	

SD, standard deviation; ^a^Include some missing values since some patients did not accept these examinations; ^b^Result of fisher’s exact test.

**Figure 2:**
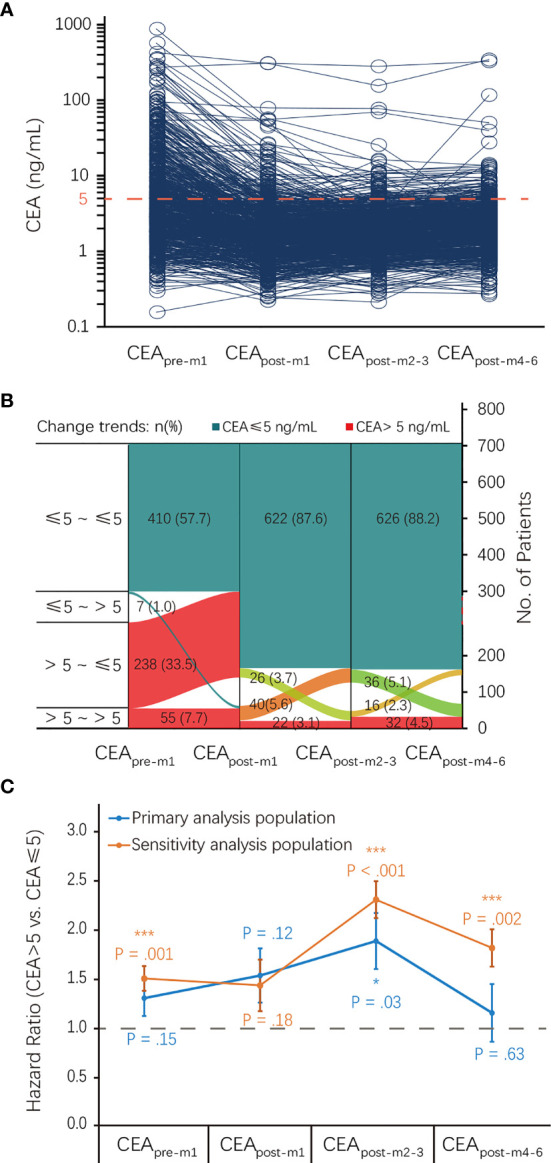
CEA status at different perioperative time points and its association with RFS. **(A)** CEA levels of each patient at different perioperative time points. **(B)** The proportion of patients with elevated CEA levels at different perioperative time points. **(C)** Association of CEA status at different perioperative time points with RFS. CEA, carcinoembryonic antigen; CI, confidence interval; HR, hazard ratio; RFS, recurrence-free survival.

The median (IQR) CEA_pre-m1_, CEA_post-m1_, CEA_post-m2–3_, and CEA_post-m4–6_ levels were 3.8 (2.0–8.8), 1.8 (1.2–2.9), 2.0 (1.3–2.9), and 2.2 (1.5–3.3) ng/mL, respectively. There were 417, 648, 662, and 642 patients with normal CEA levels before and 1, 2–3, and 4–6 months after surgery and 293, 62, 48, and 68 patients with elevated CEA levels before and 1, 2–3, and 4–6 months after surgery in the primary analysis population, respectively. The proportion of patients with elevated CEA levels at different perioperative time points showed a U-shaped curve, and the proportion observed within 2–3 months after surgery was the lowest (**Figures 2A, B**).

### Association of CEA Status at Different Perioperative Time Points With RFS and OS

There was an inverted U-shaped association between CEA status at different perioperative time points and RFS (**Figure 2C**). Univariate analysis showed that recurrence HRs in the elevated CEA_pre-m1_, CEA_post-m1_, CEA_post-m2–3_, CEA_post-m4–6_ groups were 1.30 (95% CI: 0.91–1.85), 1.53 (95% CI: 0.89–2.62), 1.88 (95% CI: 1.08–3.28), and 1.15 (95% CI: 0.91–1.85), respectively. However, this association was only significant for elevated CEA_post-m2–3_ levels (*P* = 0.03) in the primary analysis (**Table 2**). The HRs of the elevated CEA_pre-m1_, CEA_post-m1_, CEA_post-m2–3_, and CEA_post-m4–6_ groups for OS were 1.09 (95% CI: 0.60–1.97), 2.78 (95% CI: 1.34–5.79), 2.81 (95% CI: 1.25–6.30), and 3.30 (95% CI: 1.67–.536), respectively (**Table S1**).

**Table 2:** Univariate and Multivariate Analysis of 3-year Recurrence Free Survival based on Primary Analysis Population.

Variables	Univariate analysis			Multivariate analysis (M1)^b^			Multivariate analysis (M2)^c^			Multivariate analysis (M3)^d^		
	*HR*	95%CI	*P v*alue	*HR*	95%CI	*P v*alue	*HR*	95%CI	*p*-value	*HR*	95%CI	*P v*alue
CEA (>5 *vs.*≤5), ng/ml												
CEA_pre-m1_	1.30	0.91-1.85	0.15									
CEA_post-m1_	1.53	0.89-2.62	0.12									
CEA_post-m2-3_	1.88	1.08-3.28	**0.03**	1.88	1.08-3.28	**0.03**	1.91	1.210-3.34	**0.02**	1.91	1.09-3.35	**0.02**
CEA_post-m4-6_	1.15	0.65-2.05	0.63									
**Demographic variables**												
Age, years	1.00	0.98-1.01	0.74	—	—	—						
Sex (Female *vs.* Male)	1.50	1.05-2.13	**0.03**	—	—	—	1.51	1.06-2.15	**0.02**	1.51	1.05-2.15	**0.02**
BMI^a^	0.96	0.89-1.03	0.22	—	—	—						
**Clinicopathological variables**												
Primary site (Rectum *vs.* Colon)	1.52	1.06-2.17	**0.02**	—	—	—	—	—	—	1.88	1.30-2.71	**<0.001**
Tumor differentiation (Well+Moderate *vs.* Poor)^a^	0.62	0.33-1.17	0.14	—	—	—	—	—	—			
Mucinous (colloid) type (Yes *vs.* No)^a^	1.19	0.58-2.44	0.64	—	—	—	—	—	—			
T stage (reference is T1+T2)												
T3	5.63	1.39-22.82	**0.02**	—	—	—	—	—	—	7.60	1.87-30.94	**0.005**
T4	7.54	1.71-33.16	**0.008**	—	—	—	—	—	—	10.59	2.37-47.37	**0.002**
N stage (reference is N0)												
N1	1.67	1.08-2.58	**0.02**	—	—	—	—	—	—	1.63	1.05-2.52	**0.03**
N2	2.72	1.70-4.35	**<0.001**	—	—	—	—	—	—	2.59	1.61-4.14	**<0.001**
Lymph node yield (≥12 *vs.*<12)^a^	1.04	0.63-1.74	0.87	—	—	—	—	—	—			
Lymphovascular invasion (Yes *vs.* No)	1.97	1.28-3.01	**0.002**	—	—	—	—	—	—			
Perineural invasion (Yes *vs.* No)^a^	1.75	1.06-2.88	**0.03**	—	—	—	—	—	—			
Tumor deposit (Positive *vs.* Negative)^a^	2.67	1.63-4.35	**<0.001**	—	—	—	—	—	—			

HR, Hazard ratio; aInclude some missing values since some patients did not accept these examinations; bM1: Unadjusted model; cM2: Model adjusted by demographic variables; dM3: Model adjusted by demographic and clinicopathological variables.

Bold indicates P value < 0.5

Subsequently, adjusted multivariate Cox proportional hazards regression analyses showed that elevated CEA_post-m2–3_, rather than CEA_pre-m1_, CEA_post-m1_, or CEA_post-m4–6_, was an independent risk factor for recurrence, but not for OS, in the primary analysis population (**Table 2** and **Table S1**). Additionally, the adjustments resulted in a slight attenuation of the risk estimates in patients with elevated CEA_post-m2–3_, both in model 2 (elevated CEA_post-m2–3_ *vs.* normal CEA_post-m2–3_: HR, 2.38; 95% CI: 1.23–4.61) and model 3 (elevated CEA_post-m2–3_ *vs.* normal CEA_post-m2–3_: HR, 2.10; 95% CI: 1.02–4.32) (**Table 2**).

**Figure 3:**
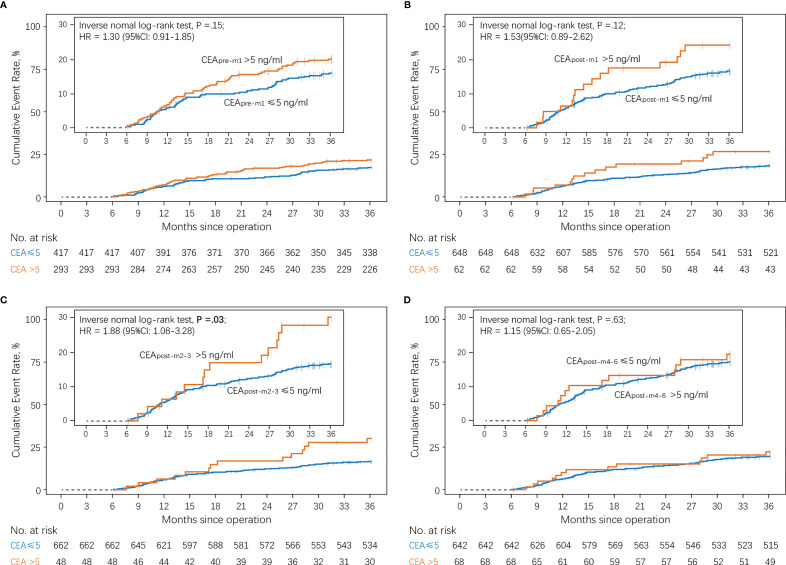
Cumulative incidence of recurrence according to serum CEA levels compared using a log-rank test **(A)** Patients with normal *vs.* elevated preoperative CEA levels (CEA_pre-m1_). **(B)** Patients with normal *vs.* elevated CEA levels 1 month after surgery (CEA_post-m1_). **(C)** Patients with normal *vs.* elevated CEA levels 2–3 months after surgery (CEA_post-m2–3_). **(D)** Patients with normal *vs.* elevated CEA levels 4–6 months after surgery (CEA_post-m4–6_). CEA, carcinoembryonic antigen.

**Figure 3** shows the cumulative incidence rates of recurrence in the normal and elevated CEA groups at different perioperative time points. There was no significant difference in the 3-year recurrence rates between those with normal and elevated CEA levels before (19.8% *vs.* 15.6%; **Figure 3A**) and 1 month (24.2% *vs.* 16.7%; **Figure 3B**) after surgery. However, patients with elevated CEA_post-m2–3_ levels showed a higher cumulative incidence rate of recurrence than patients with normal CEA_post-m2–3_ levels in the primary analysis (29.2% *vs.* 16.5%; **Figure 3C**). In contrast, no significant differences in the 3-year recurrence rates were observed between patients showing elevated and normal CEA levels 4–6 months after surgery (19.1% *vs.* 17.1%; **Figure 3D**).

### Sensitivity Analysis

The results from the sensitivity analysis are shown in **Tables S2** and **S3**. In addition to the primary analysis population, the sensitivity analysis population also included 846 patients for whom CEA_pre-m1_, CEA_post-m1_, or CEA_post-m4–6_ levels were unavailable. The results were consistent with those obtained from the primary analysis. In the sensitivity analysis population, CEA_pre-m1_ (elevated CEA_pre-m1_ *vs.* normal CEA_pre-m1_: HR, 1.50; 95% CI: 1.17–1.92) and CEA_post-m4–6_ (elevated CEA_post-m4–6_ *vs.* normal CEA_post-m4–6_: HR, 1.81; 95% CI: 1.25–2.62) were associated with significantly shorter RFS in the univariate analysis but not in the multivariate analysis.

### Subgroup Analysis

**Figure 4:**
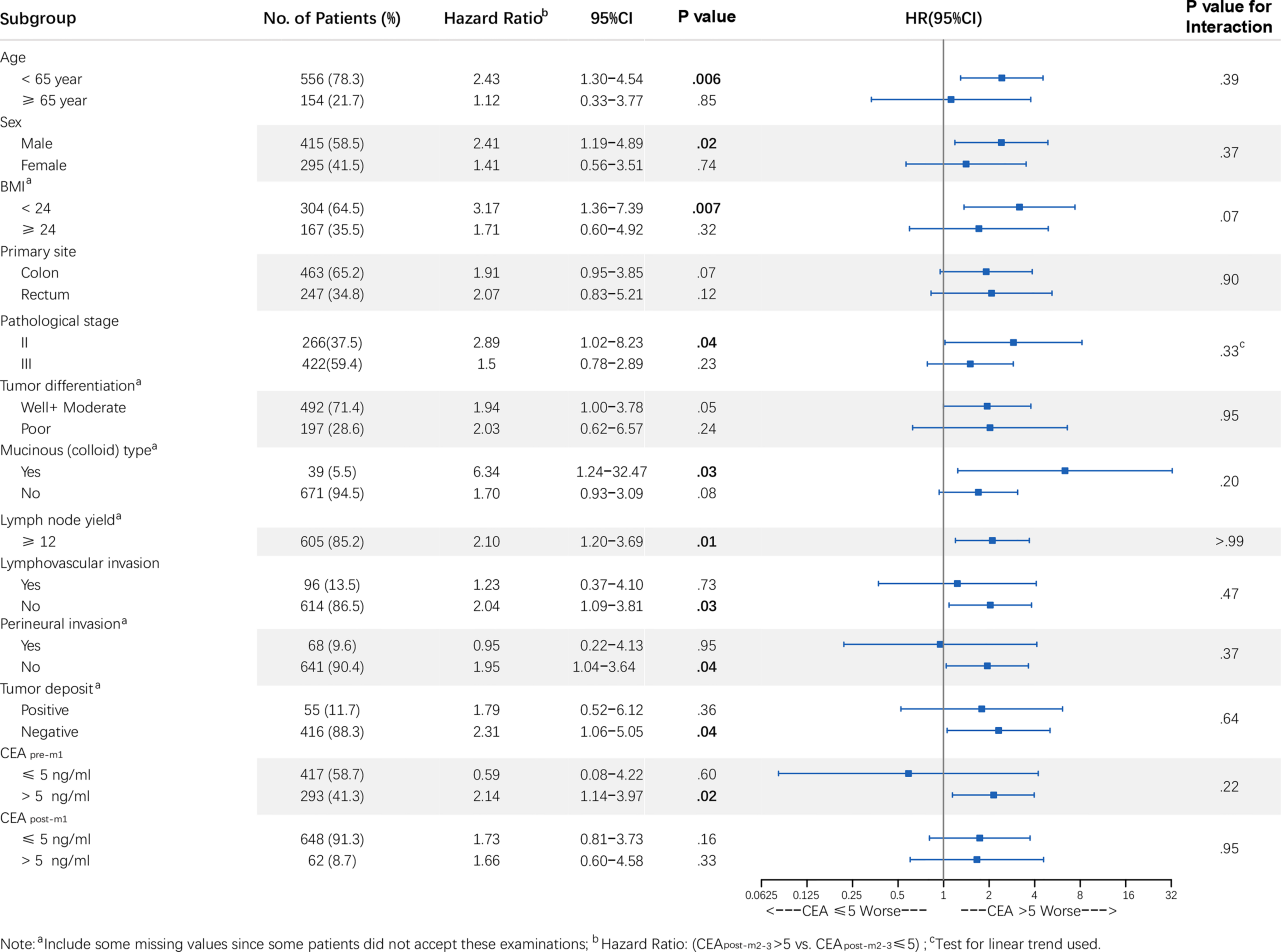
Forest plot of CEA_post-m2–3_ stratified by clinicopathological variables in the primary analysis population. Note: ^a^ Includes some missing values since some patients did not accept these examinations; ^b^ HR: (CEA >5.0 *vs.* ≤5.0 ng/mL); ^c^ Test for linear trend used. *P* values for interaction were calculated using the Cox regression model. HR and 95% CIs are provided and are visually represented by the squares and error bars. CEA, carcinoembryonic antigen; CEA_post-m2–3_, serum CEA levels 2–3 months after surgery; CI, confidence interval; HR, hazard ratio; RFS, recurrence-free survival.

Patients with elevated CEA_post-m2–3_ tended to have a higher risk of recurrence, similar to that in the overall population (**Figure 4** and **Figure S1**), in most subgroups except for among patients with normal CEA_pre-m1_. It should be noted that the RFS of the elevated and normal CEA_post-m2–3_ groups also differed significantly among patients with stage II CRC [elevated CEA_post-m2–3_ *vs.* normal CEA_post-m2–3_: HR, 2.89; 95% CI: 1.02–8.24 [primary analysis population); HR, 2.69; 95% CI: 1.34–5.38 (sensitivity analysis population)]. There were no statistically significant interactions between patients’ baseline characteristics and CEA_post-m2–3_ (all *P* > 0.05).

### Threshold Value of CEA_post-m2–3_

Patients were classified into CEA_post-m2–3_-low (≤ 5.14 ng/mL) or CEA_post-m2–3_-high (> 5.14 ng/mL) groups based on the optimal cut-off point determined by maximally selected rank statistics (**Figure S2**). And the RFS curves were statistically different (p = 0.003) when the threshold value of CEA was 5.14 ng/mL in the sensitivity analysis population (**Figure S3**).

## Discussion

Our analyses of a retrospective, multicenter longitudinal cohort of patients with stage I–III CRC who underwent curative resection showed that the association between serum CEA levels and CRC outcomes varied at different perioperative time points, and CEA_post-m2–3_ was more informative than CEA_pre-m1_, CEA_post-m1_, and CEA_post-m4–6_. Our data also showed that elevated CEA_post-m2–3_ was associated with shorter RFS. This association seemed to be independent of traditional prognostic factors and CEA levels at other perioperative time points.

We found that elevated postoperative CEA levels were more prognostic than elevated preoperative CEA levels, consistent with several previous studies (4, 10, 14–16). We also found, for the first time, that elevated CEA_post-m2–3_ is more prognostic than elevated CEA_post-m1_ and CEA_post-m4–6._ It may be postulated that the prognostic value of perioperative CEA levels is more likely to depend on the proportion of CEA reflecting the biological behavior of tumors. The elevated tumor biomarker levels are due to tumor burden and differences in the biological behavior of tumors (20). Preoperative CEA levels are both related to the tumor burden and biological behavior, while postoperative CEA levels are mainly related to biological behavior. This may be why elevated postoperative CEA levels are more prognostic than elevated preoperative CEA levels. In addition, the half-life of CEA varies from 3 to 7 days (21). Therefore, 3.0–18.0 weeks following surgery are required to allow for the clearance of CEA corresponding to tumor burden (16, 21). Interestingly, our data showed that the proportion of patients with elevated CEA levels within 2–3 months after surgery was the lowest. Together, these data indicate that the CEA level within 2–3 months after surgery may represent actual differences in the biological behavior of tumors. Hence, CEA_post-m2–3_ is more strongly associated with CRC outcomes than CEA_post-m1_ and CEA_post-m4–6_.

The sensitivity and subgroup analyses supported our findings, demonstrating that the effect estimates were robust. It is important to note that the association between CEA_post-m2–3_ and recurrence in patients with CRC may vary according to CEA_pre-m1_, with an RFS advantage seen in patients with normal CEA_post-m2–3_ and elevated CEA_pre-m1_ but not in patients with normal CEA_post-m2–3_ and CEA_pre-m1_. This suggests that elevated CEA_post-m2–3_ may not be informative when CEA_pre-m1_ is normal. Moreover, this also implies that combined use of CEA_post-m2–3_ and CEA_pre-m1_ may help clinicians in assessing the risk of recurrence better, thus allowing them to determine the optimal follow-up strategy and adjust adjuvant treatment regimens.

After subgroup analysis, our study also showed that postoperative CEA levels within 2–3 months after surgery had predictive value in patients with stage II CRC, consistent with some previous studies (11, 12). Our results confirmed that the prognostic value of serum CEA levels in patients with stage II CRC was affected by the timing of postoperative measurement. Our findings support the use of postoperative CEA measurements within 2–3 months as an indicator for the requirement of adjuvant treatment in patients with stage II CRC. And we found that the potential threshold value of CEA _post-m2–3_ was 5.14 ng/mL, which was close to 5.0 ng/mL. Besides, The CEA_post-m2–3_ had good prognostic value in OS analysis, though it was not significant in the multivariate model analysis. However, considering the clinical value of recurrence prediction, we believe that 2-3 months after surgery is the key time of perioperative serum CEA measurement.

The large size of the multicenter cohort ensured that our findings were robust when applied to different conditions, which is a major strength of our study. One limitation, however, is that different immunoassay analyzers were used for CEA measurements at the two centers. Even though harmonization of the CEA results obtained using the two immunoassay analyzers has not yet been achieved (22), the normal CEA ranges for both immunoassay analyzers are 0.0–5.0 ng/mL (17). We analyzed CEA levels as a dichotomized variable. Hence, the primary results of this study should not be affected by CEA testing methods. Another limitation is that the proportion of patients who were not treated with adjuvant chemotherapy was too low (1.5% and 8.4% in the primary and sensitivity analysis populations, respectively). Therefore, the results may not be generalizable to patients not receiving adjuvant chemotherapy. Finally, we did not control for other factors that can lead to false-positive CEA elevation (23), such as tobacco use (24), as this was challenging to accurately ascertain from the patients.

In conclusion, our study provides evidence that elevated CEA_post-m2–3_, rather than CEA_pre-m1_, CEA_post-m1_, and CEA_post-m4–6_, is associated with CRC outcomes. The optimal timing for perioperative serum CEA measurement is 2–3 months after surgery for patients with CRC, and CEA_post-m2–3_ can be used as a predictor of RFS. Our findings suggest that prolonged adjuvant chemotherapy and more frequent follow-ups should be considered to reduce the risk of relapse in CRC patients with elevated CEA_post-m2–3_.

## Data Availability Statement

The raw data supporting the conclusions of this article will be made available by the authors, without undue reservation.

## Ethics Statement

The studies involving human participants were reviewed and approved by Ethics Committee of Yunnan Cancer Hospital (KY201824). The patients/participants provided their written informed consent to participate in this study.

## Author Contributions

DY had full access to all of the data in the study and takes responsibility for the integrity of the data and the accuracy of the data analysis. Study concept and design, DY. Acquisition, analysis, or interpretation of data, ZL, ZW, XP, SY, DZ, ML, XC, QS, LC, and DY. Drafting of the manuscript, ZL, ZW, XP, and SY. Critical revision of the manuscript for important intellectual content, ZL, ZW, XP, SY, DZ, ML, XC, QS, LC, and DY. Statistical analysis, ZL, ZW, and DY. Administrative, technical, or material support, DZ, ML, XC, QS, and LC. Study supervision, DY. All authors contributed to the article and approved the submitted version.

## Funding

This study was funded by research grants from the National Natural Science Foundation of China [82001986, 81660545, 81960592, and 82073569], National Science Fund for Distinguished Young Scholars [81925023], the Outstanding Youth Science Foundation of Yunnan Basic Research Project [202101AW070001, 202001AW070021], National Key Research and Development Program of China [2017YFC1309102], the Key Science Foundation of Yunnan Basic Research [202101AS070040], the Applied Basic Research Projects of Yunnan Province, China [202001AY070001-240, 202001AY070001-242, 2019FE001-083 and 2018FE001-065], Yunnan digitalization, development and application of biotic resource [202002AA100007], the Innovative Research Team of Yunnan Province [2019-6], and the fellowship of China Postdoctoral Science Foundation [2020M670923].

## Conflict of Interest

The authors declare that the research was conducted in the absence of any commercial or financial relationships that could be construed as a potential conflict of interest.

The reviewer ZY declared a shared affiliation, with no collaboration, with XP to the handling editor at the time of the review.

## Publisher’s Note

All claims expressed in this article are solely those of the authors and do not necessarily represent those of their affiliated organizations, or those of the publisher, the editors and the reviewers. Any product that may be evaluated in this article, or claim that may be made by its manufacturer, is not guaranteed or endorsed by the publisher.

## Supplementary Material

Supplementary Figure 1 Forest plot of CEA_post-m2–3_ stratified by clinicopathological variables in the sensitivity analysis population. Note: ^a^ Includes some missing values since some patients did not accept these examinations; ^b^ HR: (CEA >5.0 *vs.* ≤5.0 ng/mL); ^c^ Test for linear trend used. *P* values for interaction were calculated using the Cox regression model. HR and 95% CIs are provided and are visually represented by the squares and error bars. CEA, carcinoembryonic antigen; CEA_post-m2–3_, serum CEA levels 2–3 months after surgery; CI, confidence interval; HR, hazard ratio; RFS, recurrence-free survival.

Supplementary Figure 2 The optimal cut-off to CEA as high-recurrence risk and low-recurrence risk patient was determined by maximally selected rank statistics method.

Supplementary Figure 3 Kaplan-Meier survival curves of RFS of CEA-low *vs* CEA-high categories in the sensitivity analysis population. CEA-low: CEA_post-m2–3_≤ 5.14 ng/mL; CEA-highd: CEA_post-m2–3_> 5.14 ng/mL
