# Supplementary figures and images for: Association Between Serum Carcinoembryonic Antigen Levels at Different Perioperative Time Points and Colorectal Cancer Outcomes
Source: Front Oncol. 2021 Oct 8;11:722883. doi: 10.3389/fonc.2021.722883 (PMC8531644; doi:10.3389/fonc.2021.722883)

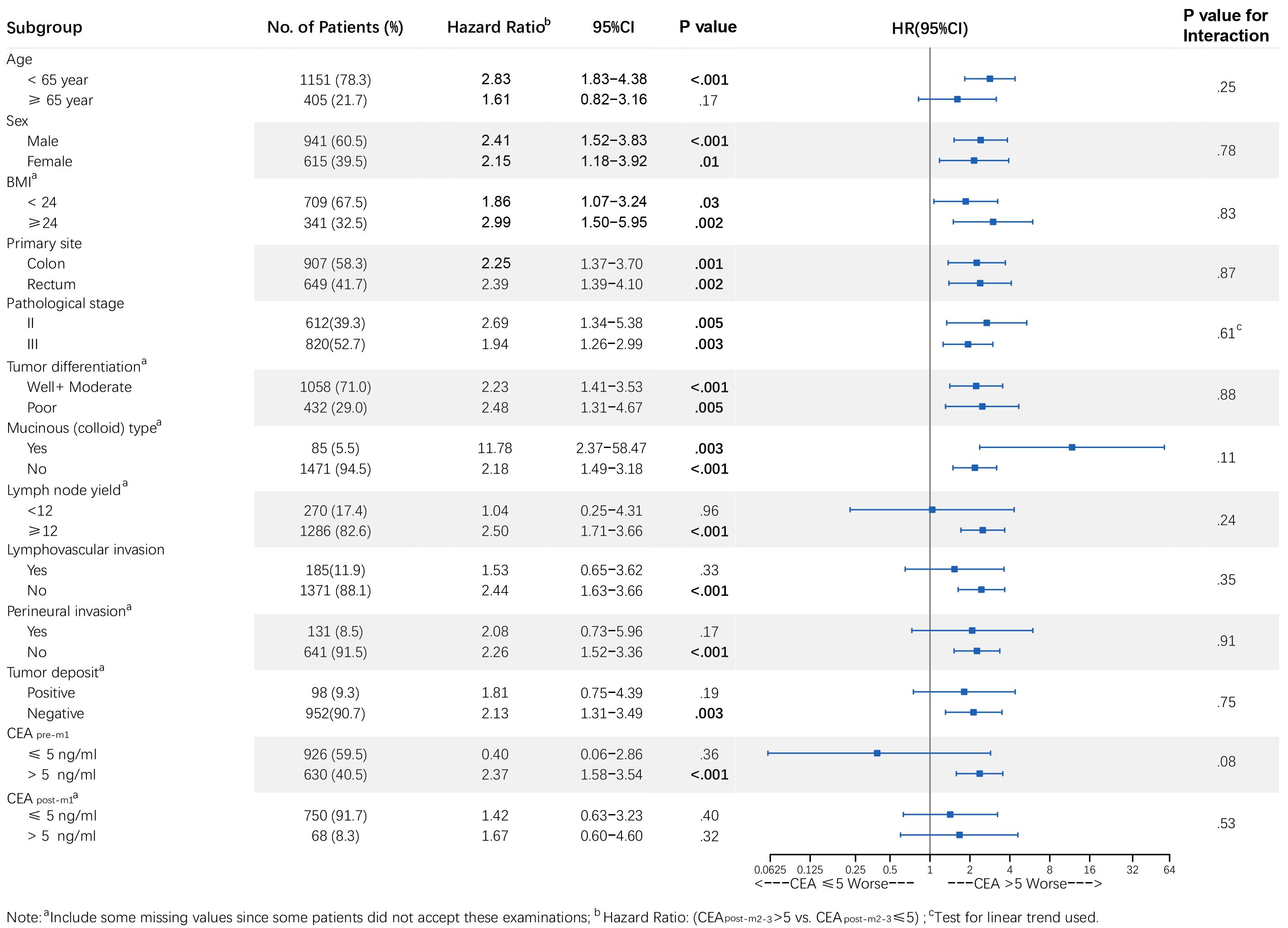

Supplement: Supplementary Figure 1 — Forest plot of CEApost-m2–3 stratified by clinicopathological variables in the sensitivity analysis population. Note: a Includes some missing values since some patients did not accept these examinations; b HR: (CEA >5.0 vs. ≤5.0 ng/mL); c Test for linear trend used. P values for interaction were calculated using the Cox regression model. HR and 95% CIs are provided and are visually represented by the squares and error bars. CEA, carcinoembryonic antigen; CEApost-m2–3, serum CEA levels 2–3 months after surgery; CI, confidence interval; HR, hazard ratio; RFS, recurrence-free survival. [file Image_1.tif]

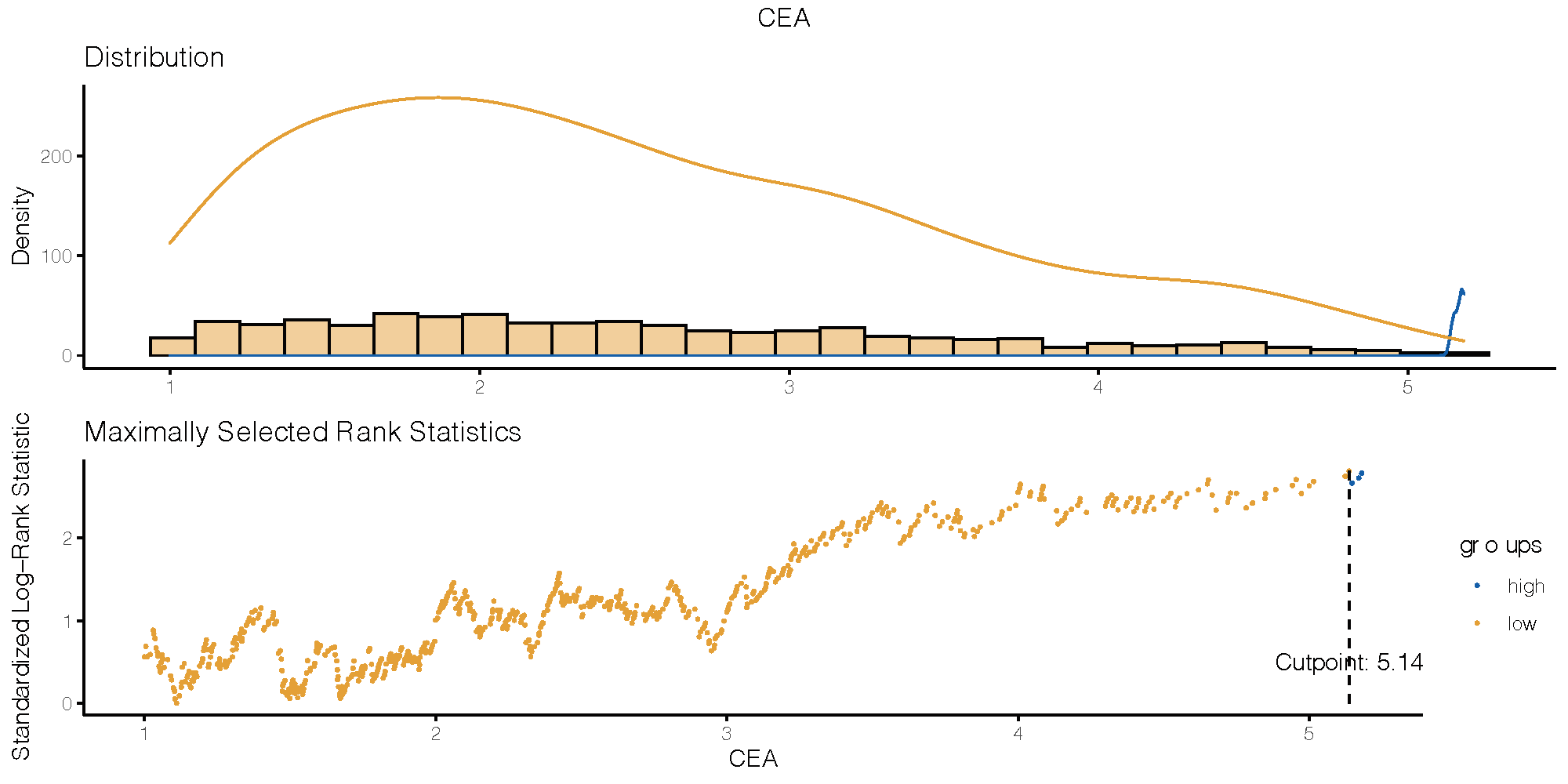

Supplement: Supplementary Figure 2 — The optimal cut-off to CEA as high-recurrence risk and low-recurrence risk patient was determined by maximally selected rank statistics method. [file Image_2.tiff]

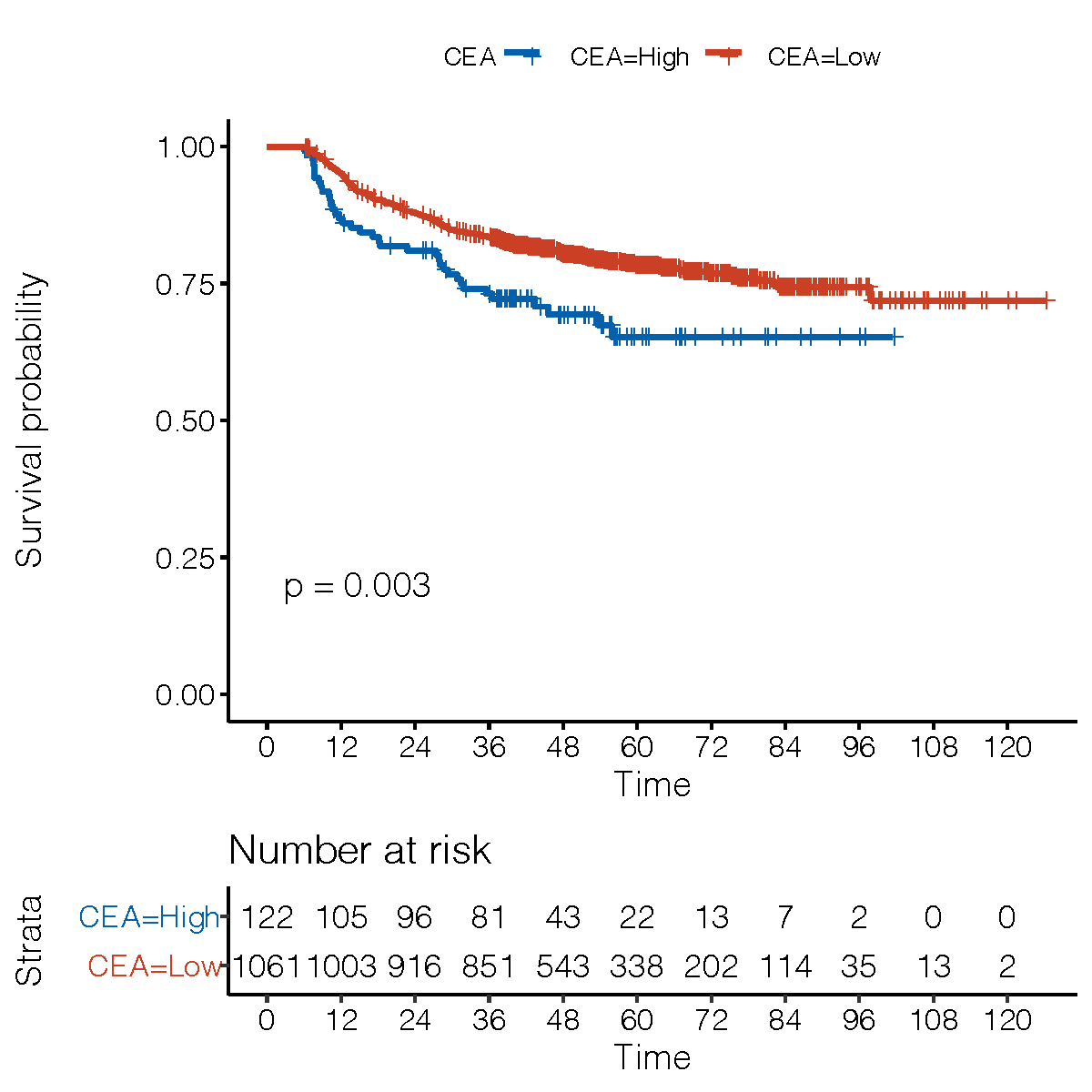

Supplement: Supplementary Figure 3 — Kaplan-Meier survival curves of RFS of CEA-low vs CEA-high categories in the sensitivity analysis population. CEA-low: CEApost-m2–3≤ 5.14 ng/mL; CEA-highd: CEApost-m2–3> 5.14 ng/mL [file Image_3.tiff]
