# Supplementary material for: Association Between Serum Carcinoembryonic Antigen Levels at Different Perioperative Time Points and Colorectal Cancer Outcomes
Source: Front Oncol. 2021 Oct 8;11:722883. doi: 10.3389/fonc.2021.722883 (PMC8531644; doi:10.3389/fonc.2021.722883)
Supplement: Supplementary file 4 [file Table_1.docx]

**Table S1. Univariate and Multivariate Analysis of 3-year Overall Survival based on Primary Analysis Population.**

| **Variables** | **Univariate analysis** | | |  | **Multivariate analysis (M1)^b^** | | |  | **Multivariate analysis (M2)^c^** | | |  | **Multivariate analysis (M3)^d^** | | |
| --- | --- | --- | --- | --- | --- | --- | --- | --- | --- | --- | --- | --- | --- | --- | --- |
|  | ***HR*** | **95%CI** | ***P v*alue** |  | ***HR*** | **95%CI** | ***P v*alue** |  | ***HR*** | **95%CI** | ***p*-value** |  | ***HR*** | **95%CI** | ***P v*alue** |
| CEA (>5 vs.≤5), ng/ml | — | — | — |  | — | — | — |  | — | — | — |  | — | — | — |
| CEA_pre-m1_ | 1.09 | 0.60-1.97 | .79 |  |  |  |  |  |  |  |  |  |  |  |  |
| CEA_post-m1_ | 2.78 | 1.34-5.79 | **.006** |  |  |  |  |  |  |  |  |  |  |  |  |
| CEA_post-m2-3_ | 2.81 | 1.25-6.30 | **.01** |  |  |  |  |  |  |  |  |  |  |  |  |
| CEA_post-m4-6_ | 3.30 | 1.67-6.53 | **<.001** |  | 3.30 | 1.67-6.53 | **<.001** |  | 3.30 | 1.67-6.53 | **<.001** |  | 2.47 | 1.23-4.97 | **.01** |
| **Demographic variables** |  |  |  |  |  |  |  |  |  |  |  |  |  |  |  |
| Age, years | 1.01 | 0.98-1.04 | .53 |  | — | — | — |  |  |  |  |  |  |  |  |
| Sex (Female vs. Male) | 1.09 | 0.60-1.98 | .78 |  | — | — | — |  |  |  |  |  |  |  |  |
| BMI^a^ | 0.99 | 0.89-1.11 | .93 |  | — | — | — |  |  |  |  |  |  |  |  |
| **Clinicopathological variables** |  |  |  |  |  |  |  |  |  |  |  |  |  |  |  |
| Primary site (Rectum vs. Colon) | 1.16 | 0.63-2.13 | .63 |  | — | — | — |  | — | — | — |  |  |  |  |
| Tumor differentiation  (Well+Moderate vs. Poor) ^a^ | 0.62 | 0.33-1.17 | .14 |  | — | — | — |  | — | — | — |  |  |  |  |
| Mucinous (colloid) type (Yes vs. No)^a^ | 2.89 | 1.22-6.84 | **.02** |  | — | — | — |  | — | — | — |  | 2.56 | 1.08-6.09 | **.03** |
| T stage (reference is T1+T2) | — | — | — |  | — | — | — |  | — | — | — |  | — | — | — |
| T3 | 3.68 | 0.51-26.86 | .20 |  | — | — | — |  | — | — | — |  | 2.13 | 0.93-4.92 | .08 |
| T4 | 6.49 | 0.78-53.95 | **.08** |  | — | — | — |  | — | — | — |  | 4.02 | 1.72-9.38 | **.001** |
| N stage (reference is N0) | — | — | — |  | — | — | — |  | — | — | — |  | — | — | — |
| N1 | 2.40 | 1.05-5.49 | **.04** |  | — | — | — |  | — | — | — |  |  |  |  |
| N2 | 4.77 | 2.06-11.05 | **<.001** |  | — | — | — |  | — | — | — |  |  |  |  |
| Lymph node yield (≥12 vs.<12)^a^ | 0.90 | 0.40-2.02 | .80 |  | — | — | — |  | — | — | — |  |  |  |  |
| Lymphovascular invasion (Yes vs. No) | 2.82 | 1.48-5.40 | **.002** |  | — | — | — |  | — | — | — |  |  |  |  |
| Perineural invasion (Yes vs. No)^a^ | 2.97 | 1.47-6.01 | **.003** |  | — | — | — |  | — | — | — |  | 2.51 | 1.23-5.15 | **0.01** |
| Tumor deposit (Positive vs. Negative)^a^ | 3.66 | 1.73-7.73 | **<.001** |  | — | — | — |  | — | — | — |  |  |  |  |

Abbreviations: HR, Hazard ratio; ^a^Include some missing values since some patients did not accept these examinations; ^b^M1: Unadjusted model; ^c^M2: Model adjusted by demographic variables; ^d^M3: Model adjusted by demographic and clinicopathological variables.
