# Supplementary material for: Association Between Serum Carcinoembryonic Antigen Levels at Different Perioperative Time Points and Colorectal Cancer Outcomes
Source: Front Oncol. 2021 Oct 8;11:722883. doi: 10.3389/fonc.2021.722883 (PMC8531644; doi:10.3389/fonc.2021.722883)
Supplement: Supplementary file 5 [file Table_2.docx]

**Table S2. Demographic and Clinicopathological Characteristics of Sensitivity Analysis Population.**

| **Characteristics** | **Total**  **(*N* =1556)** | **CEA _post-m2-3_** | | ***P v*alue** |
| --- | --- | --- | --- | --- |
|  |  | **≤5** **ng/ml (*n* =1449)** | **>5 ng/ml (*n* =107)** |  |
| Age, year |  |  |  |  |
| Mean (SD) | 56.2 (11.8) | 56.0 (11.8) | 59.2 (10.6) | **0.003** |
| Range | (18.0-90.0) | (18.0-90.0) | (28.0-78.0) |  |
|  |  |  |  |  |
| Sex, no. (%) of patients |  |  |  |  |
| Male | 941 (60.5) | 871 (60.1) | 70 (62.4) | 0.33 |
| Female | 615 (39.5) | 578 (39.9) | 37 (34.6) |  |
|  |  |  |  |  |
| BMI^a^ |  |  |  |  |
| Mean (SD) | 22.8 (3.2) | 22.8 (3.2) | 22.5 (3.3) | 0.44 |
| Range | (13.9-40.9) | (13.9-40.9) | (14.1-28.5) |  |
|  |  |  |  |  |
| Primary site, no. (%) of patients |  |  |  |  |
| Colon | 907 (58.3) | 843 (58.2) | 64 (59.8) | 0.82 |
| Rectum | 649 (41.7) | 606 (41.8) | 43 (40.2) |  |
|  |  |  |  |  |
| Tumor differentiation, no. (%) of patients |  |  |  |  |
| Well | 72 (4.6) | 71 (4.9) | 1 (0.9) | 0.12 |
| Moderate | 986 (63.4) | 911 (62.9) | 75 (70.1) |  |
| Poor | 432 (27.8) | 403 (27.8) | 29 (27.1) |  |
| Unknown | 66 (4.2) | 64 (4.4) | 2 (1.9) |  |
|  |  |  |  |  |
| Mucinous (colloid) type, no. (%) of patients^a^ |  |  |  |  |
| Yes | 85 (5.5) | 82 (5.7) | 3 (2.8) | 0.30 |
| No | 1471 (94.5) | 1367 (94.3) | 104 (93.2) |  |
|  |  |  |  |  |
| T stage, no. (%) of patients |  |  |  |  |
| T1 & T2 | 197 (12.7) | 187 (12.9) | 10 (9.4) | 0.39 |
| T3 | 1225 (78.7) | 1140 (78.7) | 85 (79.4) |  |
| T4 | 134 (8.6) | 122 (8.4) | 12 (11.2) |  |
|  |  |  |  |  |
| N stage, no. (%) of patients |  |  |  |  |
| N0 | 731 (47.0) | 695 (48.0) | 36 (33.6) | **0.002** |
| N1 | 560 (36.0) | 519 (35.8) | 41 (38.3) |  |
| N2 | 265 (17.0) | 235 (16.2) | 30 (28.0) |  |
|  |  |  |  |  |
| Lymph node yield, no. (%) of patients^a^ |  |  |  |  |
| <12 | 270 (17.4) | 257 (17.7) | 13 (12.1) | 0.18 |
| ≥12 | 1286 (82.6) | 1192 (82.3) | 94 (87.9) |  |

(continued).

| **Characteristics** | **Total**  **(*N* =1556)** | **CEA _post-m2-3_** | | ***P v*alue** |
| --- | --- | --- | --- | --- |
|  |  | **≤5** **ng/ml (*n* =1449)** | **>5 ng/ml (*n* =107)** |  |
| Pathological stage, no. (%) of patients |  |  |  |  |
| I | 124 (8.0) | 120 (8.3) | 4 (3.7) | **0.01** |
| II | 612 (39.3) | 580 (40.0) | 32 (29.9) |  |
| III | 820 (52.7) | 749 (51.7) | 71 (66.4) |  |
|  |  |  |  |  |
| Lymphovascular invasion, no. (%) of patients |  |  |  |  |
| Yes | 185 (11.9) | 166 (11.5) | 19 (17.8) | 0.07 |
| No | 1371 (88.1) | 1283 (88.5) | 88 (82.2) |  |
|  |  |  |  |  |
| Perineural invasion, no. (%) of patients^a^ |  |  |  |  |
| Yes | 131 (8.5) | 120 (8.3) | 11 (10.4) | 0.58 |
| No | 1418 (91.5) | 1323 (91.7) | 95 (89.6) |  |
|  |  |  |  |  |
| Tumor deposit, no. (%) of patients^a^ |  |  |  |  |
| Positive | 98 (9.3) | 86 (8.8) | 12 (16.4) | 0.05 |
| Negative | 952 (90.7) | 891 (91.2) | 61 (83.6) |  |
|  |  |  |  |  |
| Adjuvant chemotherapy, no. (%) of patients |  |  |  |  |
| Yes | 1426 (91.6) | 1331 (91.9) | 95 (88.8) | 0.35 |
| No | 130 (8.4) | 118 (8.1) | 12 (11.2) |  |
|  |  |  |  |  |
| Adjuvant radiotherapy, no. (%) of patients |  |  |  |  |
| Yes | 21 (1.3) | 17 (1.2) | 4 (3.7) | 0.07 |
| No | 1535 (98.7) | 1432 (98.8) | 103 (96.3) |  |

Abbreviations: SD, standard deviation; ^a^Include some missing values since some patients did not accept these examinations.
