# Supplementary material for: Association Between Serum Carcinoembryonic Antigen Levels at Different Perioperative Time Points and Colorectal Cancer Outcomes
Source: Front Oncol. 2021 Oct 8;11:722883. doi: 10.3389/fonc.2021.722883 (PMC8531644; doi:10.3389/fonc.2021.722883)
Supplement: Supplementary file 6 [file Table_3.docx]

**Table S3. Univariate and Multivariate Analysis of 3-year Recurrence Free Survival based on Sensitivity Analysis Population.**

| **Variables** | **Univariate analysis** | | |  | **Multivariate analysis (M1)^b^** | | |  | **Multivariate analysis (M2)^c^** | | |  | **Multivariate analysis (M3)^d^** | | |
| --- | --- | --- | --- | --- | --- | --- | --- | --- | --- | --- | --- | --- | --- | --- | --- |
|  | ***HR*** | **95%CI** | ***P v*alue** |  | ***HR*** | **95%CI** | ***P v*alue** |  | ***HR*** | **95%CI** | ***p*-value** |  | ***HR*** | **95%CI** | ***P v*alue** |
| CEA (>5 vs.≤5), ng/ml | — | — | — |  | — | — | — |  | — | — | — |  | — | — | — |
| CEA_pre-m1_ | 1.50 | 1.17-1.92 | **0.001** |  | 1.36 | 1.05-1.75 | **.02** |  | 1.36 | 1.05-1.75 | **.02** |  |  |  |  |
| CEA_post-m1_ ^a^ | 1.43 | 0.85-2.41 | 0.18 |  |  |  |  |  |  |  |  |  |  |  |  |
| CEA_post-m2-3_ | 2.30 | 1.59-3.31 | **<0.001** |  | 2.01 | 1.38-2.95 | **<.001** |  | 2.01 | 1.38-2.95 | **<.001** |  | 1.71 | 1.11-2.64 | **0.01** |
| CEA_post-m4-6_ ^a^ | 1.81 | 1.25-2.62 | **0.002** |  |  |  |  |  |  |  |  |  |  |  |  |
| **Demographic variables** |  |  |  |  |  |  |  |  |  |  |  |  |  |  |  |
| Age, years | 1.00 | 0.99-1.01 | 0.73 |  | — | — | — |  |  |  |  |  |  |  |  |
| Sex (Female vs. Male) | 1.13 | 0.88-1.45 | 0.35 |  | — | — | — |  |  |  |  |  |  |  |  |
| BMI ^a^ | 0.96 | 0.92-1.01 | 0.08 |  | — | — | — |  |  |  |  |  |  |  |  |
| **Clinicopathological variables** |  |  |  |  |  |  |  |  |  |  |  |  |  |  |  |
| Primary site (Rectum vs. Colon) | 1.27 | 0.99-1.62 | 0.06 |  | — | — | — |  | — | — | — |  |  |  |  |
| Tumor differentiation  (Well+Moderate vs. Poor) ^a^ | 0.74 | 0.57-0.97 | **0.03** |  | — | — | — |  | — | — | — |  |  |  |  |
| Mucinous (colloid) type (Yes vs. No)^a^ | 0.91 | 0.52--1.59 | 0.75 |  | — | — | — |  | — | — | — |  |  |  |  |
| T stage (reference is T1+T2) | — | — | — |  | — | — | — |  | — | — | — |  | — | — | — |
| T3 | 3.66 | 1.94-6.90 | **<0.001** |  | — | — | — |  | — | — | — |  | 4.00 | 1.87-8.55 | **<0.001** |
| T4 | 5.05 | 2.47-0.29 | **<0.001** |  | — | — | — |  | — | — | — |  | 3.93 | 1.54-9.98 | **0.004** |
| N stage (reference is N0) | — | — | — |  | — | — | — |  | — | — | — |  | — | — | — |
| N1 | 1.67 | 1.25-2.23 | **0.001** |  | — | — | — |  | — | — | — |  | 1.71 | 1.18-2.48 | **0.004** |
| N2 | 2.77 | 2.02-3.80 | **<0.001** |  | — | — | — |  | — | — | — |  | 3.10 | 2.12-4.54 | **<0.001** |
| Lymph node yield (≥12 vs.<12)^a^ | 1.08 | 0.77-1.51 | 0.66 |  | — | — | — |  | — | — | — |  |  |  |  |
| Lymphovascular invasion (Yes vs. No) | 1.74 | 1.26-2.41 | **0.001** |  | — | — | — |  | — | — | — |  |  |  |  |
| Perineural invasion (Yes vs. No)^a^ | 1.64 | 1.13-2.39 | **0.001** |  | — | — | — |  | — | — | — |  |  |  |  |
| Tumor deposit (Positive vs. Negative)^a^ | 2.42 | 1.67-3.50 | **<0.001** |  | — | — | — |  | — | — | — |  | 1.75 | 1.17-2.60 | **0.006** |

Abbreviations: HR, Hazard ratio; ^a^Include some missing values since some patients did not accept these examinations; ^b^M1: Unadjusted model; ^c^M2: Model adjusted by demographic variables; ^d^M3: Model adjusted by demographic and clinicopathological variables.
